# Supplementary material for: Hippocampal theta coordinates memory processing during visual exploration
Source: eLife. 2020 Mar 13;9:e52108. doi: 10.7554/eLife.52108 (PMC7069726; doi:10.7554/eLife.52108)
Supplement: Supplementary file 1. [file elife-52108-supp1.docx]

**Number of fixations for each comparison of interest.**

|  | **Pre** |  | **Post** |  |
| --- | --- | --- | --- | --- |
|  | **N_1_** | **N_2_** | **N_1_** | **N_2_** |
| **Updated vs. Repeated^*^** |  |  |  |  |
| All events | 821 | 1291 | 821 | 1291 |
| Saccade free window - 100 ms | 801 | 1246 | 811 | 1274 |
| Saccade free window - 200 ms | 558 | 835 | 597 | 909 |
| Saccade free window - 400 ms | 253 | 374 | 366 | 559 |
| **Original vs. Updated** |  |  |  |  |
| All events | 655 | 992 | 655 | 992 |
| Saccade free window - 100 ms | 639 | 969 | 646 | 979 |
| Saccade free window - 200 ms | 407 | 704 | 336 | 763 |
| Saccade free window - 400 ms | 191 | 276 | 140 | 448 |
| **Updated: Remember vs. Forgotten** |  |  |  |  |
| All events | 594 | 398 | 594 | 398 |
| Saccade free window - 100 ms | 584 | 385 | 588 | 391 |
| Saccade free window - 200 ms | 424 | 280 | 452 | 311 |
| Saccade free window - 400 ms | 172 | 104 | 265 | 183 |
| **Original vs. Repeated** |  |  |  |  |
| All events | 655 | 1136 | 655 | 1136 |
| Saccade free window - 100 ms | 639 | 1103 | 646 | 1122 |
| Saccade free window - 200 ms | 407 | 762 | 336 | 851 |
| Saccade free window - 400 ms | 191 | 307 | 140 | 498 |

**N_1_** and **N_2_** indicate the number of fixations used to compare condition 1 vs. 2

^*^only fixations to remembered locations were included in this comparison
